# Transglutaminase 2 facilitates the distant hematogenous metastasis of breast cancer by modulating interleukin-6 in cancer cells

**DOI:** 10.1186/bcr3034

**Published:** 2011-10-03

**Authors:** Keunhee Oh, Eunyoung Ko, Hee Sung Kim, Ae Kyung Park, Hyeong-Gon Moon, Dong-Young Noh, Dong-Sup Lee

**Affiliations:** 1 Laboratory of Immunology, Interdisciplinary Program of Tumor Biology, Cancer Research Institute, Seoul National University College of Medicine, 28 Yongon-dong Chongno-gu, Seoul 110-799, Korea; 2 Transplantation Research Institute, Seoul National University College of Medicine, 28 Yongon-dong Chongno-gu, Seoul 110-799, Korea; 3 Department of Surgery, Seoul National University College of Medicine, 28 Yongon-dong Chongno-gu, Seoul 110-799, Korea; 4 Dongnam Institute of Radiological & Medical Sciences, 40 Jwadong-gil Jangan-eup, Gijang-gun, Busan 619-953, Korea; 5 Department of Pathology, Hanil Hospital, 388-1 Sangmon3-dong Dobong-gu, Seoul 132-703, Korea; 6 College of Pharmacy, Sunchon National University, 255 Jungangno, Suncheon, Jeonnam 540-742, Korea

## Abstract

**Introduction:**

Inflammation has been implicated in cancer aggressiveness. As transglutaminase 2 (TG2), which has been associated with inflammatory signaling, has been suggested to play a role in tumor behavior, we propose that TG2 may be an important linker inducing interleukin (IL)-6-mediated cancer-cell aggressiveness, including distant hematogenous metastasis.

**Methods:**

To investigate the role for TG2 and IL-6, TG2-knocked-down and IL-6-knocked-down cancer cells were generated by using shRNA. Human breast cancer cell xenograft model in highly immunocompromised mice and human advanced breast cancer primary tumor tissue microarrays were used in this study.

**Results:**

IL-6 production in human breast cancer cells was dependent on their TG2 expression level. *In vitro *tumor-sphere formation was dependent on TG2 and downstream IL-6 production from cancer cells. Primary tumor growth in the mammary fat pads and distant hematogenous metastasis into the lung was also dependent on TG2 and downstream IL-6 expression levels. The effect of TG2 expression on human breast cancer distant metastasis was investigated by analyzing a tissue microarray of primary tumors from 412 patients with their clinical data after 7 years. TG2 expression in primary tumor tissue was inversely correlated with recurrence-free survival (*P *= 0.019) and distant metastasis-free survival (DMFS) (*P *= 0.006) in patients with advanced breast cancer. Furthermore, by using public datasets that included a total of 684 breast cancer patients, we found that the combined high expression of TG2 and IL-6 was associated with shorter DMFS, compared with the high expression of IL-6 only (*P *= 0.013).

**Conclusions:**

We provide evidence that TG2 is an important link in IL-6-mediated tumor aggressiveness, and that TG2 could be an important mediator of distant metastasis, both in a xenograft animal model and in patients with advanced breast cancer.

## Introduction

Immune/inflammatory responses have largely been considered a key protective mechanism of hosts against growing tumor cells [1]. However, inflammation is now also recognized to be important in the pathogenesis of many types of malignancies [2]. Persistent *Helicobacter pylori *infection is associated with gastric cancer [3]. Viral infections lead to chronic inflammation and are responsible for the majority of hepatocellular carcinomas [4]. Obesity also promotes chronic inflammation and results in a substantial increase in cancer risk in the liver [5] and pancreas [6].

Inflammation thus triggered by infection, obesity, or the tumor itself recruits inflammatory cells to the tumor-stroma interface, and these cells, together with the tumor cells, generate a microenvironment capable of driving tumor progression [7]. The concerted action of inflammatory cytokines, together with oxidative stress and hypoxia in the tumor environment converge to activate nuclear factor (NF)-κB in cancer cells [8]. NF-κB signaling has been implicated in several cancer cell behaviors, including initiation, promotion, survival, malignant conversion, invasion, and metastasis [9].

Interleukin (IL)-6 is an important downstream effector of NF-κB. High serum IL-6 levels correlate with poor disease outcome and reduced clinical prognosis in patients with breast, lung, and liver cancer [10,11] and with cancer formation in a murine colitis-associated colon cancer model [12]. IL-6, produced from bone marrow-derived cells, promotes growth of tumor-initiating cells during early tumorigenesis and protects these cells from apoptosis [12]. Furthermore, IL-6 produced in epithelial cancer cells themselves plays an important role in tumor growth and metastasis, in an autocrine and/or paracrine manner. IL-6 signaling pathways in epithelial cancer cells have also been linked to *in vivo *aggressiveness by affecting epithelial-to-mesenchymal conversion [13,14] or conferring the cancer stem cell-like properties of these cells [15]. The molecular links leading to IL-6 production in epithelial cancer cells, which are correlated with distant metastasis and cancer stem cell-like properties, are currently under active investigation. Noninfectious stimuli activating the IL-6 signaling pathway lead to fibrosis through transglutaminase 2 (TG2) in pulmonary epithelial cells (unpublished data). As fibrosis and invasion of cancer have common characteristics [16], we propose that TG2 expressed in epithelial cancer cells might provide a similar connection.

TG2 has been implicated in the drug resistance and survival of cancer cells by modulating caspase-3 and NF-κB activity [17-20] and in the *in vitro *migration and invasion of tumor cells through increased cell attachment via β-integrins and fibronectins with extracellular TG2 [21-23] and increases in matrix metalloprotease-2 (MMP-2) expression [24]. Peritoneal spreading of ovarian cancer cells [25] and tumor growth of pancreatic cancer [26] also parallel TG2 expression levels. Recently, correlation of TG2 expression and epithelial-to-mesenchymal transition and invasion of cancer cells were reported for both breast and ovarian cancer cells [27,28]. However, a direct link between TG2 expression of primary tumors and distant hematogenous metastasis in animal models and cancer patients and cancer stem cell-like properties, which are related to cancer cell aggressiveness, has not been made.

In this study, we demonstrated that TG2 expression levels are correlated with cancer cell aggressiveness, and that the size and efficiency of tumor sphere formation was correlated with TG2 expression levels and was dependent on TG2-mediated IL-6 secretion in breast cancer cells. With highly immunocompromised mice, we evaluated the critical role of TG2 and downstream IL-6 in the distant metastasis of breast cancer cells. Moreover, TG2 expression in primary tumor tissue was inversely correlated with recurrence-free survival and distant metastasis-free survival (DMFS) in patients with breast cancer, evaluated from a tissue microarray of primary tumors. Furthermore, by using public datasets that included a total of 684 breast cancer patients, we found that the expression of TG2 and IL-6 was correlated in breast cancer patients and that the combined high expression of TG2 and IL-6 was related to a poor DMFS outcome in breast cancer. Thus, in this study, we provide evidence that TG2 is an important link in IL-6-mediated tumor aggressiveness and that TG2 could be an important mediator of distant metastasis in both a xenograft animal model and in patients with advanced breast cancer.

## Materials and methods

### Cell lines and TG2- and IL-6-knockdown

The human breast cancer cell lines were obtained from the American Type Culture Collection (Manassas, VA, USA). TG2-knocked-down MDA-MB-231 cells (shTG2_MB231) were established by using the short-hairpin RNA (shRNA) vector for TG2. The target sequence for TG2 was 5'-GGGCGAACCACC TGAACAA-3' [29]. In brief, MDA-MB-231 cells were co-transfected with the pcDNA3.1 plus pSUPER plasmid containing shRNA for TG2 by using Promofectin (PromoKine, Heidelberg, Germany), according to the manufacturer's instructions. Control cells were transfected with the pcDNA3.1 vector only. TG2-knockdown in selected clones was shown by Western blot analysis. IL-6 production in MDA-MB-231 cells was inhibited by using the IL-6 shRNA vector (shIL-6). Cells transfected with the shIL-6 vector were cultured in the presence of G418, and IL-6-knocked-down MDA-MB-231 (shIL-6_MB231) clones were selected by performing an IL-6 enzyme-linked immunosorbent assay (ELISA).

### Western blot analysis

Cells were harvested in a lysis solution containing 1% NP40, a protease inhibitor cocktail, and a phosphatase inhibitor (Sigma-Aldrich///). Proteins (10 μg) were separated with SDS-PAGE, and transferred polyvinylidene difluoride membranes were probed with appropriate antibodies: anti-TG2 (Neomarkers, Fremont, CA, USA), anti-β-actin (Sigma-Aldrich), anti-Bcl-2, and anti-cIAP2 (Santa Cruz Biotechnology, Santa Cruz, CA, USA).

### ELISA and MTT assay

In total, 1 × 10^4 ^cells were applied on 24-well plates and allowed to adhere overnight. Then the medium was replaced, and cells were permitted to grow for 24, 48, or 72 hours. To inhibit TG2 activity, some cells were treated with cysteamine (Sigma-Aldrich). Supernatants were collected and assayed to quantify soluble IL-6 production by ELISA. All assays were performed in triplicate and were repeated 2 or 3 times under independent conditions. Data are presented as mean ± SD.

### Sphere formation

Cells grown in an anchorage-independent condition were trypsinized and resuspended in serum-free medium consisting of a 1:1 mixture of F-12 Nutrient Mixture (Ham's F-12) and DMEM, supplemented with 20 ng/ml epithelial growth factor (Invitrogen, Carlsbad, CA, USA), 20 ng/ml basic fibroblast growth factor (Millipore, Billerica, MA, USA), 0.5 nM hydrocortisone (Sigma-Aldrich), B-27 supplement (GIBCO), and 2 mM L-glutamine (GIBCO///). Cells were applied on nonadherent 96-well plates (Corning, Corning, NY, USA) at 10, 100, or 1,000 cells/100 μl/well. To inhibit TG2 activity, cells were treated with cysteamine///. The medium was replaced every 3 days. Visible spheres were counted under a microscope on day 8 after plating.

### Animals and xenografts

All mice were bred and maintained in specific pathogen-free conditions at the animal facility of Seoul National University College of Medicine. All animal experiments were performed with the approval of the institutional animal care and use committee of Seoul National University.

To assess tumorigenicity of the cancer cells, human breast cancer MDA-MB-231 cells were injected into the mammary fatpads into 8-week-old NOD/scid/IL-2Rγ^-/- ^(NOG) mice (Jackson Laboratory, Bar Harbor, ME, USA). Six weeks after the injection, the mice were killed, and primary tumor masses in the fatpads for breast cancer and lung were fixed in 4% paraformaldehyde for 24 hours. Sections (4 μm) were stained with hematoxylin and eosin. The number of tumor masses in the liver and lung were counted under a dissecting microscope.

### Tissue microarray and immunohistochemistry

To investigate the clinical significance of TG2 expression in human breast cancer tissue, TG2 expression was evaluated by immunohistochemistry in a tissue microarray. The tissue microarray was constructed from representative paraffin-embedded tumor tissues by an experienced breast pathologist. The array consisted of two cores per patient. The tissue microarray slides were stained with anti-TG2 (1:100, Neomarkers////). Antigen retrieval was facilitated by microwaving the tissue for 21 minutes, and the remainder of the staining procedure followed the standard avidin-biotinylated peroxidase complex method by using a commercial immunohistochemical kit (Envision-Kit; Dako, Carpinteria, CA, USA). The TG2 expression level in tumor tissue was determined by comparison with the TG2 expression in normal endothelial cells. Immunohistochemical staining was interpreted and categorized with an arbitrary semiquantitative scale as 0 (negative staining), 1+ (equivalent to normal endothelial cell expression), and 2+ (more than normal endothelial cell expression) by a pathologist. Tumors showing TG2 expression (score, 2+) were categorized into a strong-TG2 group, and tumors showing 0 or 1+ TG2 expression, into a negative/weak-TG2 group for further statistical analysis. This study was approved by the Seoul National University Hospital institutional review board.

### Analysis of public human breast cancer gene expression datasets

We collected three public mRNA expression datasets comprising a total of 684 breast cancer patients (GEO accession numbers: [GSE7390], [GSE11121], and [GSE2034]) [30-32]. The raw data were normalized by using the RMA approach, and final log_2_-transformed data were used for further analyses. Clinical data were downloaded from the same site. The level of TG2 expression was calculated by averaging the expression values of three probe sets (211003_x_at, 211573_x_at, and 216183_at). Survival was analyzed based on distant metastasis-free survival (DMFS) by using Kaplan-Meier curves and the log-rank test.

### Statistical analyses

The IL-6 production and cell-viability results are expressed as mean ± SD. A two-tailed Student *t *test was used to compare measurements taken from the two samples. For *in vivo *experiments, differences in the numbers of tumor masses and tumor volume were analyzed by using a two-tailed Student *t *test. A two-tailed χ^2 ^test and Student *t *test were used to compare nominal and continuous variables of the TG2 expression groups in the human breast cancer tissue microarray. Univariate survival analysis was performed with a Kaplan-Meier curve and the log-rank test. The Cox proportional hazard model was used for the survival analysis. All *P *values < 0.05 were deemed to indicate statistical significance. The SPSS software (ver. 11.0; SPSS Inc., Chicago, IL, USA) was used for all statistical analyses.

## Results

### TG2 expression levels correlated with IL-6 production

**Figure 1:**
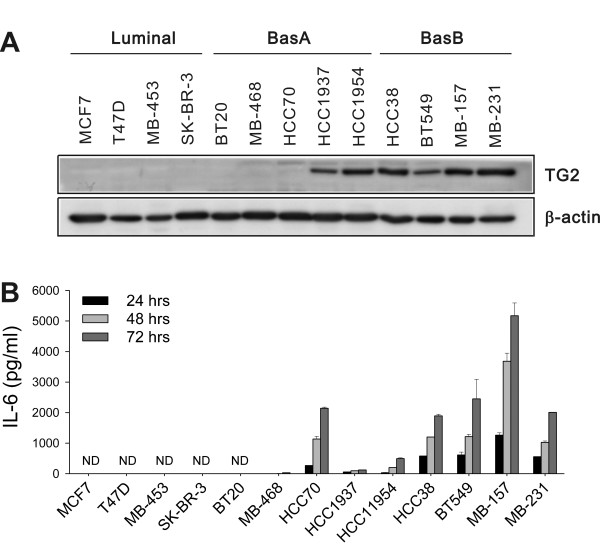
**TG2 expression levels in cancer cells correlated with IL-6 production**. **(a) **TG2 expression in the 13 indicated human breast cancer cell lines was analyzed with Western blot. **(b) **IL-6 levels in culture supernatants of breast cancer cells were determined with enzyme-linked immunosorbent assay (ELISA). 1 × 10^4 ^cells were applied on 24-well plates and allowed to adhere overnight. Then the medium was replaced, and cells were permitted to grow for 24, 48, or 72 h. Data represent mean ± SD, based on three independent experiments using samples from triplicate cell cultures. IL-6, interleukin 6; SD, standard deviation; TG2, transglutaminase 2.

To evaluate the correlation between TG2 expression and biologic characteristics of cancer cells, we first analyzed TG2 expression in various human breast cancer cells encompassing luminal, basal A, and basal B types, according to their gene-expression profiles [33,34]. TG2 expression was confined to some basal A and all basal B cells tested, the most biologically aggressive types, but not in ER^+ ^or ER^+/-^Her-2^+ ^luminal types (Figure 1a). Next, we measured IL-6 production from breast cancer cell culture supernatants and found that cells expressing a high level of TG2 produced a large amount of IL-6. Some breast cancer cells neither expressed TG2 nor produced IL-6 (Figure 1b).

### TG2-knockdown reduced IL-6 production in breast cancer cells

**Figure 2:**
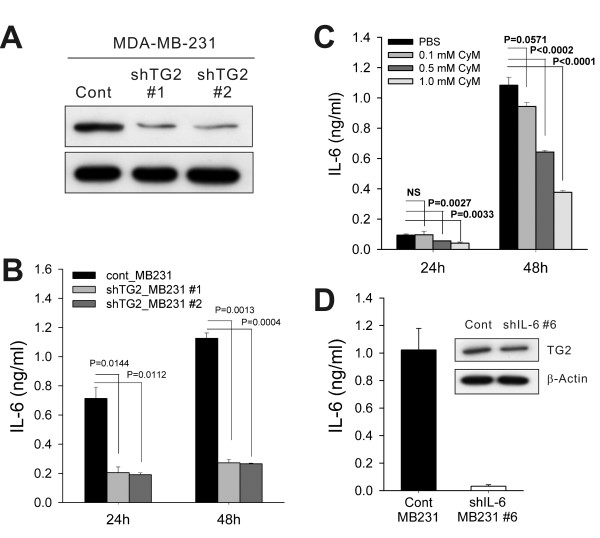
**TG2-knocked-down breast and ovarian cancer cells secreted less IL-6**. **(a) **MDA-MB-231 human breast cancer cells were stably transfected with shRNAs targeting TG2. The efficiency of knockdown was assessed with Western blot. **(b) **IL-6 levels in the culture supernatants of control and TG2-knocked-down MDA-MB-231 cells were determined with ELISA. **(c) **Effect of the TG2 inhibitor cysteamine (CyM) on IL-6 secretion in control MDA-MB-231 cells was determined with ELISA. **(d) **MDA-MB-231 cells were stably transfected with shRNAs targeting IL-6. The efficiency of knockdown was verified by measuring IL-6 levels in the culture supernatants. TG2 expression of control and IL-6-knocked-down MDA-MB-231 cells was analyzed with Western blot. **(b-d) **1 × 10^4 ^cells were applied on 24-well plates and allowed to adhere overnight. Then the medium was replaced, and cells were permitted to grow for 24 or 48 h **(b, c) **or for 48 h only **(d)**. Data represent mean ± SD, based on three independent experiments using samples from triplicate cell cultures. IL-6, interleukin 6; SD, standard deviation; TG2, transglutaminase 2.

To evaluate whether modulation of TG2 expression levels in the given cancer cells leads to a change in IL-6 production, we compared IL-6 levels in control empty vector-transfected TG2 high-expressing cells, MDA-MB-231 (cont_MB231), and their TG2-knocked-down cells by using shRNA vectors (shTG2_MB231; Figure 2a). shTG2_MB231 showed reduced IL-6 production, compared with cont_MB231 (Figure 2b). A causal link between TG2 activity and IL-6 production was confirmed by using the TG2 inhibitor cysteamine. Inhibition of TG2 enzymatic activity reduced IL-6 production from TG2-high-expressing cells (Figure 2c). These findings indicate that TG2 is a key regulator of IL-6 production in breast cancer cells. We also established IL-6-knocked-down breast cancer cells (shIL-6_MB231) by using the shIL-6 vector. The shIL-6_MB231 showed reduced IL-6 secretion, but revealed TG2 expression levels comparable to those of control cells (Figure 2d). Thus IL-6 production was downstream of TG2 activity in the breast cancer cells.

### Anchorage-independent survival of cancer cells was dependent on TG2-mediated IL-6 expression

**Figure 3:**
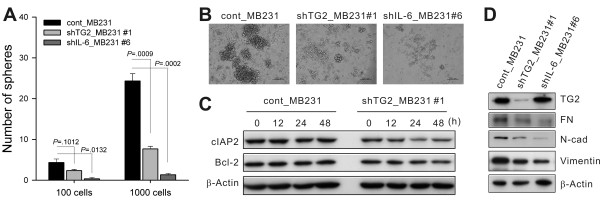
**TG2-mediated IL-6 allows anchorage-independent survival of cancer cells**. **(a) **Control and TG2-knocked-down MDA-MB-231 cells were cultured in serum-free medium consisting of a 1:1 mixture of Ham F-12 and DMEM, supplemented with growth factors. Visible spheres were counted under a microscope on day 8 after plating. Data represent mean ± SD based on three independent experiments using samples from triplicate cell cultures. **(b) **Phase-contrast microscopy of day 8 spheres generated from the MDA-MB-231 cell line. Data shown are representatives of three independent experiments. **(c) **cIAP2 and Bcl-2 expression was analyzed with Western blot after culture for the indicated time in sphere medium. **(d) **TG2, fibronectin (FN), N-cadherin (N-Cad), and vimentin expression of control, TG2-knocked-down, and IL-6-knocked-down MDA-MB-231 cells analyzed with Western blot. IL-6, interleukin 6; SD, standard deviation; TG2, transglutaminase 2.

TG2 expression endows aggressiveness and invasiveness to cancer cells through multiple effector pathways [35]. As tumor sphere formation and tumor cell aggressiveness are correlated [36] and IL-6 in tumor cells triggers malignant features in tumor spheres [15], we evaluated whether TG2 and downstream IL-6 were involved in tumor-sphere formation *in vitro*. Sphere formation decreased markedly both in TG2-knocked-down and IL-6-knocked-down breast cancer cells (shTG2_MB231#1 and shIL-6_MB231#6, respectively) compared with the control empty vector-transfected cells (Figure 3a and 3b). The numbers and sizes of the spheres were reduced both in TG2-knocked-down and IL-6-knocked-down cells (Figure 3a and 3b). Compared with TG2-knocked-down cells (shTG2_MB231#1), control cancer cells (cont_MB231) cultured in sphere medium expressed more antiapoptotic molecules, such as cIAP2 and Bcl-2 (Figure 3c). These data indicate that TG2 supported cell survival in an anchorage-independent condition. In addition, control cancer cells expressed more epithelial-to-mesenchymal transition (EMT)-related molecules such as N-cadherin and vimentin, compared with either TG2-knocked-down (shTG2_MB231#1) or IL-6-knocked-down cells (shIL-6_MB231#6) (Figure 3d).

### TG2 and downstream IL-6 facilitate tumor growth and distant liver and lung metastasis of breast cancer cells

Previous results demonstrated that high TG2 expression resulted in high IL-6 expression, which was correlated with increased anchorage-independent growth. To evaluate the role of TG2 and downstream IL-6 in tumor cell behavior *in vitro*, the effect of TG2 and its downstream IL-6 on *in vivo *tumor spreading and distant metastasis was evaluated by using highly immunocompromised NOD/scid/IL-2Rγ chain-deficient (NOG) mice, which completely lack NK activity, as well as T- and B-cell activity, as recipients [37].

**Figure 4:**
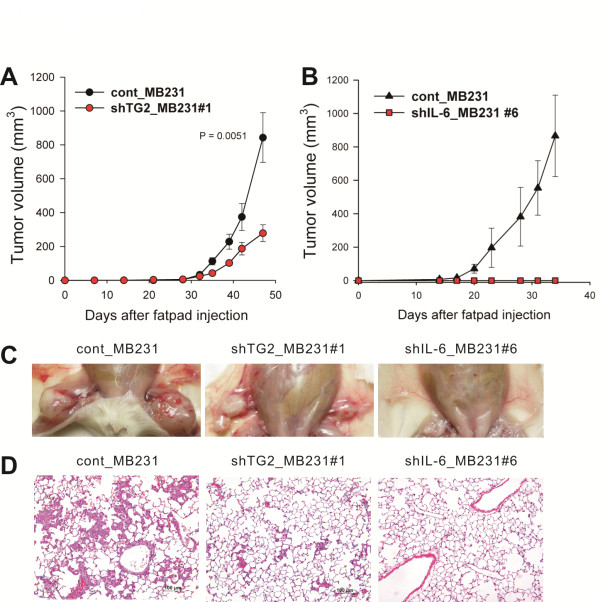
**TG2-knocked-down and IL-6-knocked-down breast cancer cells revealed decreased tumor formation and distant metastasis**. **(a) **Control and TG2-knocked-down MDA-MB-231 cells (2 × 10^5 ^cells/each mouse) were injected in the fatpads of NOG mice. The growth of primary tumors was measured. **(b) **Control and IL-6-knocked-down MDA-MB-231 cells (2 × 10^5 ^cells/each mouse) were injected in the fatpads of NOG mice. The growth of primary tumors was measured. **(a, b) **Data are given as mean ± SD of 14 mice for each group from two independent experiments. **(c, d) **At 45 days after the inoculation of cancer cells, the mice were killed, and the primary tumor masses in the fatpads and distant metastases in the liver, lung, and other organs were analyzed. **(c) **Tumor formation in the fatpads of mice xenografted with MDA-MB-231 cells. **(d) **Lung sections from mice xenografted with MDA-MB-231 cells were obtained and stained with hematoxylin and eosin (original magnification, ×200). **(c, d) **Data shown are representatives of each group. IL-6, interleukin 6; NOG, NOD/scid/IL-2Rγ chain-deficient; SD, standard deviation; TG2, transglutaminase 2.

Breast cancer cells with high TG2 levels (cont_MB231) showed rapid growth of primary tumor masses in the mammary fatpads compared with TG2-knocked-down cells (shTG2_MB231#1, Figure 4a and 4c) and IL-6-knocked-down cells (shIL-6_MB231#6, Figure 4b and 4c). Notably, all the mice injected with IL-6-knocked-down cells showed no tumor mass formation at all in the fatpads up to 45 days after tumor inoculation (Figure 4b and 4c). More interestingly, our highly immunocompromised NOG mice inoculated with TG2-high-expressing cont_MB231 showed spontaneous distant hematogenous metastases, revealing multiple visible tumor masses in the lung parenchyma (Figure 4d). In contrast, much reduced lung metastasis was noted from mice injected with TG2-knocked-down cells (shTG2_MB231#1), and no metastasis, with IL-6-knocked-down cells (shIL-6_MB231#6) (Figure 4d).

### TG2 expression predicted recurrence and metastasis in patients with breast cancer

**Figure 5:**
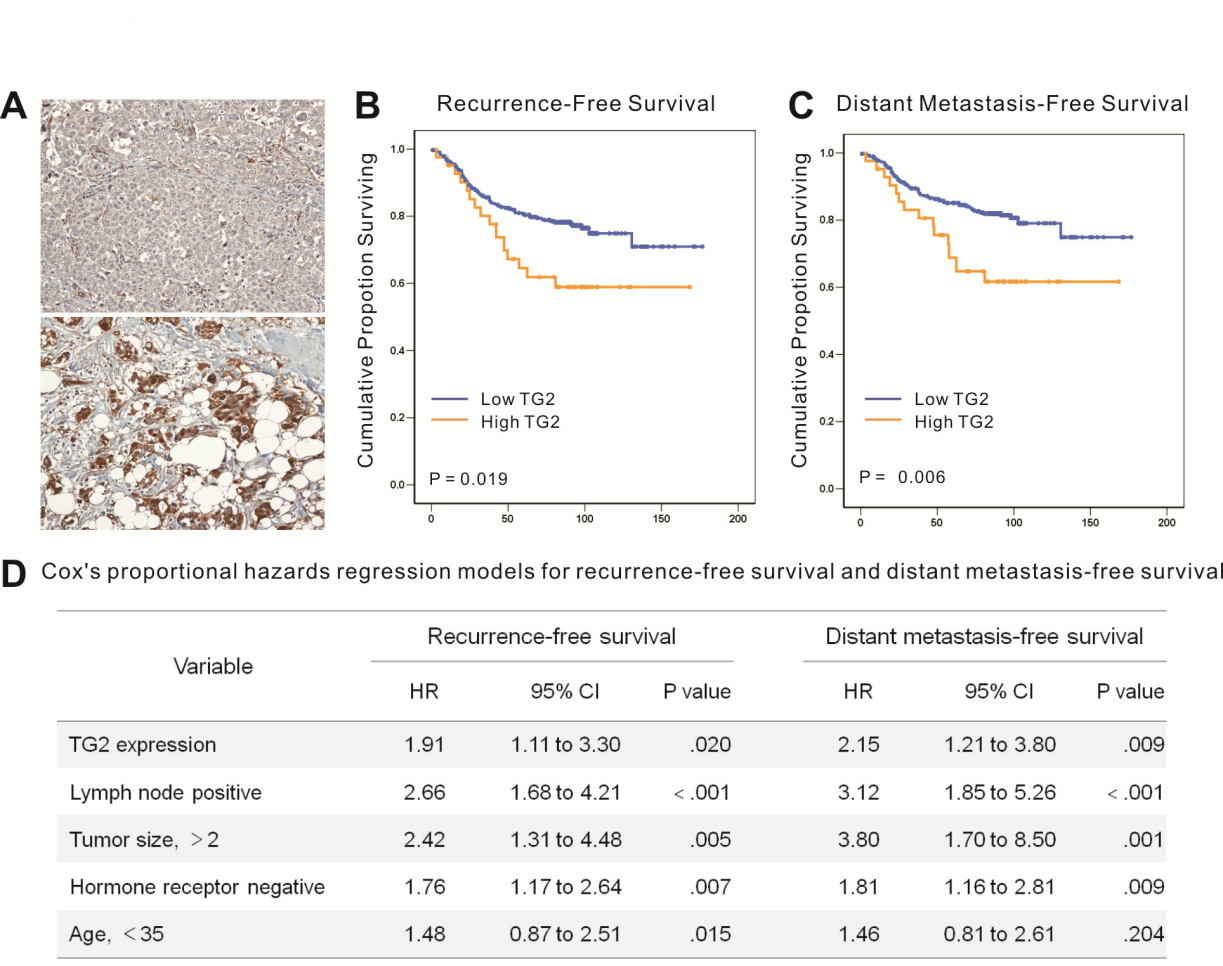
**TG2 expression predicts recurrence and metastasis in patients with breast cancer**. TG2 expression was analyzed with immunohistochemistry in a tissue microarray of primary tumors from patients with advanced breast cancer. **(a) **Immunohistochemical staining for TG2 in the primary tumors of patients with breast cancer showing negative (upper) and strong expression (lower) (×200). **(b) **Recurrence-free survival and **(c) **distant metastasis-free survival according to TG2 expression adjusted for tumor size (≤2 cm versus > 2 cm), histologic grade (1, 2 versus 3), lymph node status (positive versus negative), and hormone-receptor status (positive versus negative). The mean follow-up duration for patients after surgery was 83.6 ± 29.8 months. **(d)**/////Table 1. Cox's proportional hazards regression models for recurrence-free and distant metastasis-free survival. TG2, transglutaminase 2.

The clinical implications of TG2 expression in human breast cancer primary tumor tissue were examined with a tissue microarray from 443 patients with operable breast cancer treated at Seoul National University Hospital between February 2000 and December 2002. Among them, 31 patients were excluded because their microarray core did not contain breast tumor tissue or the tissue was detached during the immunostaining process, leaving 412 patients for the final analysis. The mean follow-up duration was 83.6 months (± 29.8 months). Forty-two (10.2%) patients showed strong TG2 expression, compared with control endothelial cells (Figure 5a). The TG2 expression level was not significantly associated with known breast cancer prognostic factors, such as tumor size, lymph node metastasis, age, or estrogen-receptor status (Additional file 1 Table S1). However, patients with TG2-upregulated tumors had significantly shorter recurrence-free survival and distant metastasis-free survival (*P *= 0.019 and *P *= 0.006, respectively; Figure 5b and 5c) than those who did not. The TG2 expression level remained an independent prognostic factor predicting recurrence and distant metastasis after adjusting for known prognostic factors by using a Cox proportional hazard model (Figure 5d). These findings suggest that TG2 in human breast cancer primary tumor may play a critical role in cancer cell metastasis and recurrence.

**Figure 6:**
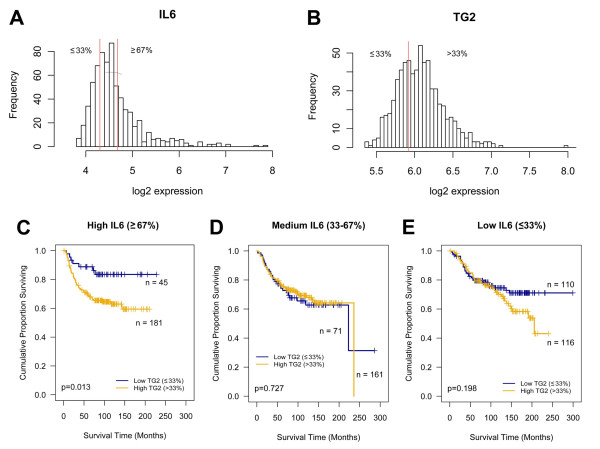
**Expression of TG2 and IL-6 in patients with breast cancer and their relation to distant metastasis-free survival**. In total, 684 human breast cancer gene-expression profiles were obtained from three public datasets, and the association between the expression of TG2/IL-6 and distant metastasis-free survival (DMFS) was analyzed. The distributions of the log_2 _expression levels of IL-6 **(a) **and TG2 **(b)**. DMFS according to TG2 expression in the patients with high **(c)**, medium **(d)**, and low **(e) **IL-6 expression. IL-6, interleukin 6; TG2, transglutaminase 2.

In the previous section, we demonstrated that the expression of TG2 and IL-6 was correlated and that high TG2 expression was associated with a high proportion of distant metastasis and larger primary tumors in a mouse xenograft model. To confirm this relation in patient clinical data, public datasets that included a total of 684 breast cancer patients were collected, and the association between DMFS and TG2/IL-6 expression was investigated. First, the patients were stratified into three groups based on the level of IL-6 expression: high (patients with ≥67% IL-6 expression), medium (33~67%), and low IL-6 (≤33%) (Figure 6a). Subsequently, the patients in each group were further stratified into two groups according to the level of TG2 expression: low TG2 included patients with ≤33% expression level of TG2, and high TG2 included all other patients (Figure 6b). Finally, the survival analysis revealed that in the high IL-6 group, most of the patients expressed high levels of TG2 (*n *= 181), and only 20% of the patients (*n *= 45) expressed low levels of TG2 (Figure 6c). In addition, high TG2 was associated with a significantly (*P *= 0.013) shorter DMFS compared with low TG2 in this group. However, no significant survival differences were detected between the high- and low-TG2 groups in the low- or medium-IL-6 groups (Figure 6d and 6e). Conversely, the proportion of patients with low TG2 expression increased as the expression of IL-6 decreased (Figure 6c through 6e), indicating that the expression of TG2 and IL-6 is also correlated in breast cancer patients. In conclusion, combined high expression of TG2 and IL-6 was related to a poor DMFS outcome in breast cancer.

## Discussion

Tumor metastasis is one of the most serious and relevant problems in tumor treatment. The formation of metastases is a complex process, which requires the action of various effectors not yet completely identified. In this study, we found that TG2 expression in tumor cells is essential for tumor growth, invasion, and distant hematogenous metastasis. Furthermore, a tissue microarray analysis of 412 patients with advanced breast cancer and a 7-year follow-up revealed that TG2 expression in primary tumor tissue is correlated with recurrence-free survival and distant metastasis-free survival.

Cancer-sphere formation is an *in vitro *assay to detect a cancer cell subpopulation that shows anchorage-independent growth *in vitro *and *in vivo *tumorigenesis [15], called cancer-initiating cells. For distant metastasis, detached cells from the tumor microenvironments enter the circulation. The survival of circulating cells is affected by inflammatory cytokines, such as tumor necrosis factor alpha (TNF-α) and IL-6 [38]. TNF-α has controversial effects in cancer pathogenesis, including induction of apoptosis or EMT of tumor cells [39,40]. IL-6 is produced from epithelial tumor cells themselves as well as stromal cells, and cancer-derived IL-6 induces a cancer stem cell-like phenotype in these cells [15]. Moreover, sustained activation of STAT3, a downstream IL-6 signaling mediator, is important for stem cell self-renewal [41,42].

In this study, *in vitro *tumor-sphere formation of breast cancer cells was largely dependent on TG2 expression and was inhibited by a TG2 enzymatic inhibitor. IL-6 production from cancer cells was dependent on TG2 expression, and tumor-sphere formation was correlated with TG2-dependent IL-6 production in cancer cells, indicating that the effect of TG2 on tumor-sphere formation is mediated through downstream IL-6 production. A correlation between *in vitro *tumor-sphere formation and *in vivo *tumorigenesis was confirmed in breast cancer cells, and TG2 expression and downstream IL-6 production were profoundly correlated with primary tumor growth of the human breast cancer cells in the mammary fatpads.

*In vivo *tumor invasion and metastasis of epithelial cancer cells has recently been associated with EMT of tumor epithelial cells [43]. TGF-β and downstream Snails, Twists, and ZEBs are associated with EMT phenotypes [44]. IL-6 has also been implicated in the induction of the breast cancer cell EMT phenotype [14], and recent reports suggest the role of TG2 in the EMT phenomena of both breast and ovarian cancer cells [27,28]. Thus, TG2 and downstream IL-6 may be important in the EMT of epithelial cancer cells and associated with *in vivo *invasiveness and distant metastasis of these cells.

With the advantage of using highly immunocompromised mice, we constructed a spontaneous distant metastasis model by using TG2-high-expressing MDA-MB-231 human breast cancer cells implanted in the orthotopic mammary fatpads. Forty-five days after implantation of control MDA-MB-231 cells, all the mice showed visible lung metastases. Lung metastases were decreased in the mice xenografted with TG2-knocked-down cells, and none of the mice xenografted with IL-6-knocked-down cells showed distant metastases. Thus TG2 and downstream IL-6 produced from epithelial breast cancer cells facilitated distant hematogenous lung metastases of these cells.

In this study, the TG2 and downstream IL-6 signaling pathway may act as a convergence site for EMT and cancer-initiating cell phenotypes, leading to enhanced local tumor growth and distant hematogenous metastasis. Thus, targeting either TG2 or downstream IL-6 would be a very efficient strategy to inhibit primary tumor growth and distant hematogenous metastasis of breast cancer cells. Complete inhibition of primary tumor growth and distant lung metastasis by IL-6-knockdown compared with some residual tumor growth and metastases by TG2-knockdown in the breast cancer cells. In addition to the IL-6 signaling pathway, TG2 can directly modulate the extracellular matrix (ECM) structure [45], activate focal adhesion kinase via binding with integrins [35], and activate growth factors such as TGF-β [46], a key inducer of EMT, buried in the ECM networks, and thus may affect a broader aspect of tumor biology.

The clinical significance of TG2 expression in breast cancer has been reported; TG2 is upregulated in epithelial and stromal cells of tumor tissue [47], and lymph node metastatic tumors show significantly higher TG2 expression levels than do primary tumors in the same patients [48]. In patients with ovarian cancer, TG2 is upregulated in cancer tissue compared with the normal counterpart [25], and TG2 overexpression is associated with high tumor stage [49]. However, a direct clinical correlation between TG2 expression in primary tumor and distant metastasis in patients is not available.

In this study, we assessed the correlation between TG2 expression in primary breast cancer tissue and distant metastasis by analyzing a tissue microarray of 412 patients and their metastasis status for approximately 7 years (mean follow-up duration, 83.6 ± 29.8 months). Although the overall survival rate of breast cancer patients did not correlate with TG2 expression levels, patients with TG2-upregulated tumors showed significantly shorter relapse-free survival and distant metastasis-free survival, after adjusting for known prognostic factors such as tumor size, lymph node metastasis, age, and hormone-receptor status. Thus, TG2 was an independent prognostic factor predicting recurrence and distant metastasis in patients with breast cancer. As increased TG2 expression has also been reported in metastatic tumor tissue [48], we propose that TG2 is an important mediator and/or prognostic marker for distant metastasis, affecting both cancer-initiating cell-like and EMT phenotypes. Thus, intervention specific to TG2 in epithelial cancer cells could provide a promising means of controlling tumor metastasis.

## Conclusions

We have observed that differential TG2 expressions of cancer cells were dictated into their IL-6 production, and TG2 and downstream IL-6 production from cancer cells wase correlated with their capacity for tumor-sphere formation *in vitro*. Primary tumor growth in the mammary fatpads and distant hematogenous metastasis of breast cancer cells into the lung was also dependent on TG2 and downstream IL-6 expression levels. We also found that TG2 expression in primary tumor tissue is inversely correlated with recurrence-free survival (*P *= 0.019) and distant metastasis-free survival (*P *= 0.006) in patients with advanced breast cancer. Thus, TG2 expression in cancer cells is an important link in IL-6-mediated tumor aggressiveness, and TG2 could be an important mediator of distant metastasis, both in a xenograft animal model and in patients with advanced breast cancer.

## Abbreviations

EMT: epithelial-to-mesenchymal transition; IL-6: interleukin-6; MMP-2: matrix metalloprotease-2; NF-κB: nuclear factor (NF)-κB; NOG: NOD/scid/IL-2Rγ chain-deficient; TG2: transglutaminase 2; TNF-α: tumor necrosis factor alpha.

## Competing interests

The authors declare that they have no competing interests.

## Authors' contributions

KO conducted most of the experiments and contributed to the planning and analysis. EK and HGM generated TMA and conducted clinical follow-up. HSK scored TMA. DYN helped design the study. DSL was responsible for the overall direction of the project. All authors have read and approved the manuscript for publication.

## Supplementary Material

Additional file 1 **Table S1**. Association between TG2 expression and other clinicopathologic factors. Click here for file
