# Supplementary material for: Transglutaminase 2 facilitates the distant hematogenous metastasis of breast cancer by modulating interleukin-6 in cancer cells
Source: Breast Cancer Res. 2011 Oct 3;13(5):R96. doi: 10.1186/bcr3034 (PMC3262209; doi:10.1186/bcr3034)
Supplement: Additional file 1 — Table S1. Association between TG2 expression and other clinicopathologic factors. [file bcr3034-S1.DOCX]

**Supplementary Tables**

**Supplementary Table 1. Association between TG2 expression and other clinicopathological factors**

| Characteristics | Group | TG2 negative, *n* (%) | | TG2 strong, *n* (%) | | *P* value |
| --- | --- | --- | --- | --- | --- | --- |
| Age (y) | ＜35 | 46 | (12.5) | 2 | (4.8) | .203 |
|  | ≥35 | 322 | (87.5) | 40 | (95.2) |  |
| Tumor Size (cm) | ≤2 | 131 | (35.4) | 10 | (23.8) | .133 |
|  | ＞2 | 239 | (64.6) | 32 | (76.2) |  |
| Lymph node metastasis | Negative | 189 | (51.1) | 22 | (52.4) | .873 |
|  | Positive | 181 | (48.9) | 20 | (47.6) |  |
| Histological grade | 1,2 | 208 | (56.4) | 22 | (52.4) | .622 |
|  | 3 | 161 | (43.6) | 20 | (47.6) |  |
| Estrogen receptor | Negative | 173 | (47.3) | 21 | (50.0) | .737 |
|  | Positive | 193 | (52.7) | 21 | (50.0) |  |
| Progesterone receptor | Negative | 248 | (67.8) | 24 | (57.1) | .167 |
|  | Positive | 118 | (32.2) | 18 | (42.9) |  |
| Her-2 | Negative | 284 | (77.8) | 30 | (73.2) | .501 |
|  | Positive | 81 | (22.2) | 11 | (26.8) |  |
| Bcl-2 | Negative | 142 | (39.2) | 14 | (34.1) | .527 |
|  | Positive | 220 | (60.8) | 27 | (65.9) |  |
| Ki-67 | ＜10% | 225 | (63.6) | 29 | (72.5) | .263 |
|  | ≥10% | 129 | (36.4) | 11 | (27.5) |  |
| P53 | ＜50% | 276 | (76.0) | 33 | (80.5) | .524 |
|  | ≥50% | 87 | (24.0) | 8 | (19.5) |  |
| Chemotherapy | No | 60 | (16.2) | 4 | (9.5) | .271 |
|  | Yes | 310 | (83.8) | 38 | (90.5) |  |
| Hormonal Therapy | No | 16 | (7.6) | 3 | (13.0) | .745 |
|  | Yes | 194 | (92.4) | 20 | (87.0) |  |
